# A rare case of osteogenesis imperfecta (OI): clinical image

**DOI:** 10.11604/pamj.2022.41.9.32859

**Published:** 2022-01-05

**Authors:** Prasad Pramod Dhage, Chaitanya Ajay Kulkarni

**Affiliations:** 1 Department of Community Health Physiotherapy, Ravi Nair Physiotherapy College, Datta Meghe Institute of Medical Sciences, Sawangi, Wardha, Maharashtra, India

**Keywords:** Osteogenesis Imperfecta, multiple fractures, low bone mineral density

## Image in medicine

A 19-year-old male patient admitted to hospital having experienced multiple fractures over a past decade, predominantly due to falling down or over-exertion. The clinical signs and radiological features, such as repeated fractures of tibia and fibula with humerus, blue sclera and low bone mineral density levels, all led to the diagnosis of moderate form of type 1 osteogenesis imperfecta (A,B). The patient began treatment with regular intake of calcium that is 1000 milligram per day, and adequate intake of vitamin D that is 800 milligram per day and intravenous methylprednisolone 50 milligram, with mild to moderate level of physiotherapy rehabilitation program. Following one month of treatment the symptoms and quality of life of the patient improved. The patient appeared to be the rare genetic case of type 1 osteogenesis imperfecta (C,D).

**Figure 1:**
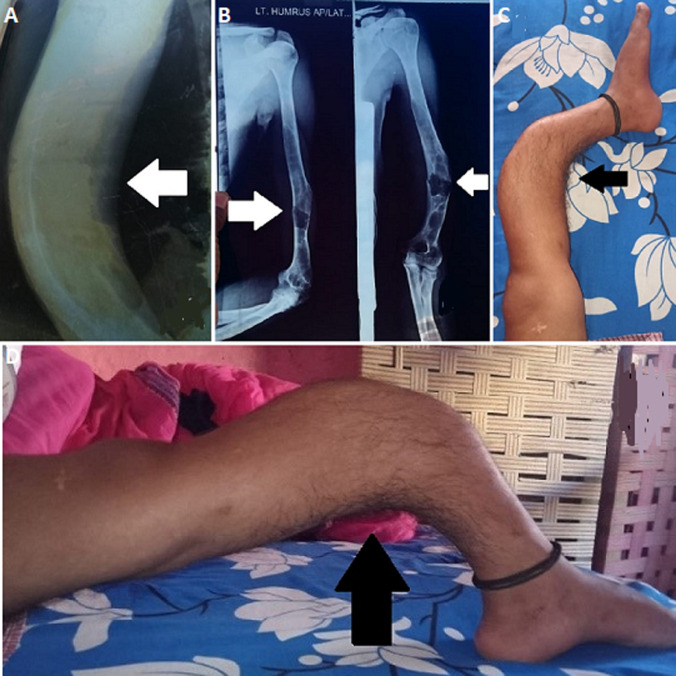
A) left lower limb (tibia and fibula) showing osteogenesis imperfecta; B) left fractures of shaft of humerus; (C,D) left lower limb (tibia and fibula) showing osteogenesis imperfect

